# Microneedling-assisted delivery of metformin versus tranexamic acid in treating melasma: a randomized controlled study

**DOI:** 10.1186/s40001-025-03032-1

**Published:** 2025-08-18

**Authors:** Abdelshakour Al Mohammady, Ahmed S. Kadah, Mohamed A. Mahran, Mohamed L. Elsaie

**Affiliations:** 1 Department of Dermatology, Venereology and Andrology, Faculty of Medicine, Al-Azhar University, Cairo, Egypt; 2 Department of Dermatology, Faculty of Medicine for Girls, Al-Azhar University, New Damietta, Egypt; 3 Department of Dermatology, Medical Research and Clinical Studies Institute, National Research Centre, Giza, Egypt

**Keywords:** Melasma, Microneedling, Metformin, Tranexamic acid

## Abstract

**Background:**

Melasma is a common, relapsing hyperpigmentation disorder with limited long-term treatment success. Recent advances suggest that microneedling may enhance the transdermal delivery and efficacy of topical agents. This study compares the clinical outcomes of microneedling-assisted delivery of topical metformin and tranexamic acid (TXA) in the treatment of facial melasma, with modified Kligman’s formula as a control.

**Objectives:**

To evaluate and compare the efficacy, safety, and patient satisfaction of microneedling with topical metformin versus TXA in melasma patients. Methods: Forty-five female patients with facial melasma were randomized into three equal groups. Group A received microneedling with topical metformin; Group B received microneedling with topical TXA; Group C applied modified Kligman’s formula nightly. Treatments were conducted over 8 weeks. Outcomes included changes in modified Melasma Area and Severity Index (mMASI), patient satisfaction scores, pain levels, and adverse events. Results: All groups showed significant mMASI reductions. Group B achieved the greatest reduction (mean 45.3%) followed by Group C (38.2%) and Group A (22.1%) (*p* < 0.001). Satisfaction scores were highest in Group B, with 33.3% reporting marked improvement. Minimal adverse events were reported across all groups, with no serious side effects. Pain scores were comparable between microneedling groups.

**Conclusion:**

Microneedling with topical TXA demonstrated superior efficacy and patient satisfaction compared to metformin and Kligman’s formula. While topical metformin showed some potential, its formulation and clinical utility require further study. Microneedling offers a promising approach to improve treatment outcomes in melisma.

**Graphical Abstract:**

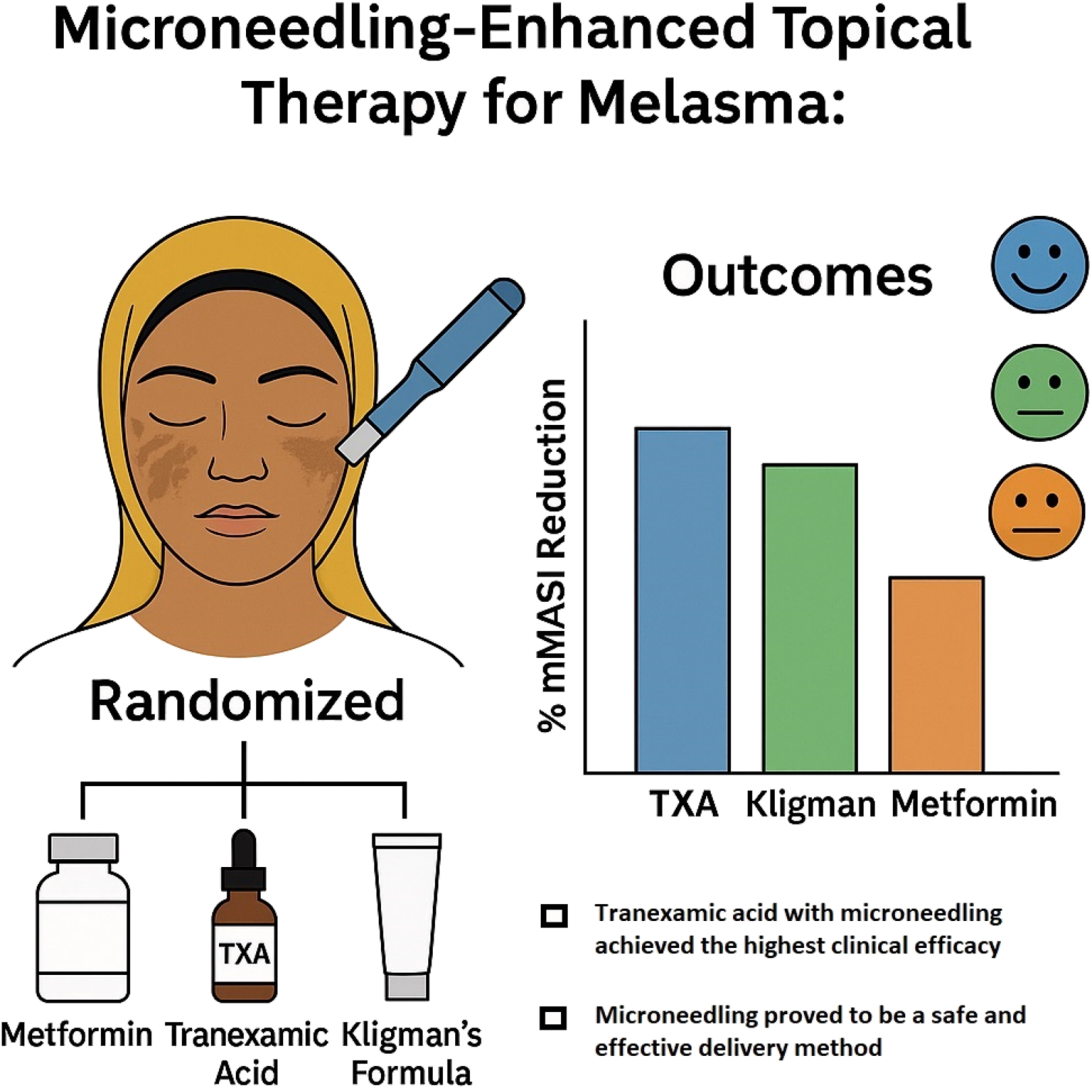

## Introduction

Melasma is a common acquired pigmentary disorder characterized by symmetrical, blotchy, brownish facial hypermelanosis that primarily affects women, particularly those of reproductive age and individuals with darker skin types (Fitzpatrick III–VI) [1].

The pathogenesis of melasma is multifactorial, involving ultraviolet (UV) radiation exposure, hormonal influences, genetic predisposition, oxidative stress, vascular abnormalities, and inflammation. Despite the availability of numerous therapeutic options—including topical bleaching agents, chemical peels, and laser therapy—the condition remains therapeutically challenging due to its chronicity and high recurrence rates [2].

Microneedling has gained increasing attention as a minimally invasive procedure that enhances transdermal drug delivery through controlled epidermal microinjuries, which improve percutaneous absorption while stimulating collagen production and dermal remodeling. When combined with topical agents, microneedling may increase treatment efficacy by facilitating deeper skin penetration [3].

Tranexamic acid (TXA), a synthetic antifibrinolytic agent, has demonstrated promising results in melasma treatment through inhibition of plasminogen activation, thereby reducing melanocyte stimulation and neovascularization. Topical and intradermal forms of TXA have shown clinical efficacy with favorable safety profiles [3]. More recently, metformin, a well-known antidiabetic agent, has emerged as a novel depigmenting option due to its inhibitory effect on melanogenesis via the AMP-activated protein kinase (AMPK)/MITF pathway [4].

This study aimed to evaluate and compare the therapeutic efficacy and safety of microneedling with topical metformin versus microneedling with topical TXA, in comparison with modified Kligman’s formula—a widely used combination therapy.

## Patients and methods

### Study design

This was a randomized, prospective, parallel-group clinical trial conducted to compare the efficacy and safety of microneedling combined with topical metformin solution versus microneedling combined with topical tranexamic acid (TXA) solution and a control group treated with topical modified Kligman’s formula in patients with facial melasma. The study took place at the Dermatology Outpatient Clinic, Al-Hussein University Hospital, Cairo, Egypt. Ethical approval was obtained from the institutional review board, and all procedures conformed to the Declaration of Helsinki guidelines. Written informed consent was obtained from each participant.

### Participants

The study included 45 female patients aged 20–50 years with clinically diagnosed facial melasma. Eligibility criteria included stable melasma of at least 3 months’ duration, Fitzpatrick skin types III or IV, and no use of melasma treatment or depigmenting agents within the preceding 3 months. Exclusion criteria comprised pregnancy, lactation, recent isotretinoin use, photosensitizing drug intake, systemic diseases such as diabetes or lupus, active facial dermatoses, history of keloids, or any contraindication to microneedling or topical therapies.

### Randomization and group allocation

Simple randomization was performed using a computer-generated table. Allocation concealment was ensured through opaque, sequentially numbered sealed envelopes prepared by a third-party researcher and opened only after enrollment. The treatment groups were as follows:

- Group A: Microneedling followed by topical metformin solution.
- Group B: Microneedling followed by topical TXA solution.
- Group C: Topical modified Kligman’s formula (hydroquinone 4%, tretinoin 0.05%, mometasone 0.1%) once daily.

### Baseline assessment

All patients underwent a comprehensive baseline evaluation that included full dermatological examination, clinical photography, dermoscopy (DermLite DL4), and Wood’s lamp examination to classify melasma type (epidermal, dermal, or mixed). Melasma distribution (centrofacial, malar, mandibular), disease duration, and family history were recorded. Fitzpatrick skin type classification was determined using standard questionnaires.

### Treatment procedure

For microneedling sessions in Groups A and B, the face was cleansed and disinfected, and topical anesthesia (lidocaine–prilocaine cream) was applied for approximately 45 min. Microneedling was performed using a derma pen device (Dr. Pen Ultimate A6) equipped with a 36-needle cartridge set at 1.5 mm depth. The pen was passed in vertical, horizontal, and oblique directions until pinpoint bleeding was noted. Four sessions were conducted biweekly over eight weeks.

- Metformin Solution (Group A): A freshly prepared topical solution was applied immediately after microneedling. It was formulated using metformin hydrochloride combined with a polyethylene glycol-based cream base, including acetyl alcohol, parabens, and liquid paraffin. The topical metformin solution was freshly prepared by dissolving metformin hydrochloride (15% w/w) in a hydrophilic cream base composed of polyethylene glycol-6 and polyethylene glycol-32, cetyl alcohol, methylparaben, propylparaben, and liquid paraffin. The mixture was homogenized for uniformity and adjusted to a pH of 6.2 to optimize skin tolerability.
- TXA Solution (Group B): TXA (500 mg/5 mL) was diluted with normal saline to a 50 mg/mL concentration. Approximately 1–2 mL was applied after each microneedling session.
- Kligman’s Formula (Group C): Patients received topical modified Kligman’s formula without microneedling once daily at bedtime for 8 weeks.

All patients were instructed to avoid direct sun exposure and apply a broad-spectrum sunscreen (SPF ≥ 50) twice daily, along with a moisturizer. Patients in Groups A and B were advised to use topical fusidic acid cream for 3 days post-microneedling to prevent infection.

### Outcome measures

The primary outcome was the change in the modified Melasma Area and Severity Index (mMASI) from baseline to 1 month after the last treatment. The mMASI score evaluates four facial areas (forehead, right malar, left malar, and chin) using scores for area and darkness, with a total score ranging from 0 to 24.

Secondary outcomes included:

- Patient satisfaction, assessed via a 4-point quartile scale (0: < 25%, 1: 25–49%, 2: 50–74%, 3: ≥ 75% improvement).
- Pain score, measured using a 10-point numeric rating scale immediately after each session.
- Adverse events, including erythema, edema, PIH, scarring, or infection.
- Dermoscopy findings, evaluated pre- and post-treatment for pigmentation pattern changes.

### Statistical analysis

Data analysis was performed using IBM SPSS version 20. Continuous variables were expressed as mean ± standard deviation (SD) and compared using ANOVA or Kruskal–Wallis tests. Categorical variables were assessed using the Chi-square test or Fisher’s exact test. A p-value < 0.05 was considered statistically significant. All analyses were conducted on an intention-to-treat basis. Non-parametric Kruskal–Wallis tests were also conducted for variables where non-normal distribution was suspected (e.g., mMASI reduction % in Group A), confirming the robustness of the results.

## Results

### Baseline characteristics

The mean age of patients was 36.3 ± 8.1 years in Group A, 35.7 ± 9.4 years in Group B, and 37.1 ± 10.1 years in Group C, with no significant difference between groups (p = 0.925). Fitzpatrick skin type III was the most common in all groups (Group A: 66.7%, Group B: 60%, Group C: 46.7%), followed by type IV, and there were no significant differences in skin type distribution (p = 0.529) Table 1.

**Table 1:** Comparison between the studied groups as regard baseline data

	Group A (Metformin) (n = 15)			Group B (Tranexamic)(n = 15)			Group C (Kligman) (n = 15)			Test		P
Age										F = 0.078		0.925
Range	23 – 49			24 – 50			20 – 50					
Mean ± SD	36.33 ± 8.1			35.73 ± 9.41			37.07 ± 10.11					
Skin type	No	%		No	%		No	%		χ^2^ = 1.275	0.529	
type III	10	66.7		9	60.0		7	46.7				
type IV	5	33.3		6	40.0		8	53.3				
Disease duration										H = 0.319	0.853	
Range	3 – 44			3 – 38			3 – 50					
Median (IQR)	9 (3 – 13.5)			14 (3 – 27.5)			9 (3 – 23.5)					
Family history	No		%	No		%	No		%			
−ve	10		66.7	8		53.3	12		80.0	χ^2^ = 2.400	0.301	
+ ve	5		33.3	7		46.7	3		20.0			
Clinical type												
Centrofacial	3		20.0	1		6.7	2		13.3	χ^2^ = 3.630	0.458	
Malar	7		46.7	12		80.0	9		60.0			
Mandibular	5		33.3	2		13.3	4		26.7			
Pattern of melasma												
Epidermal	10		66.7	9		60.0	9		60.0	χ^2^ = 0.189	0.910	
Mixed	5		33.3	6		40.0	6		40.0			

*IQR* interquartile range, *χ*^*2*^ Chi square test, *H* Kruskal Wallis test, *SD* Standard deviation, *F* one-way ANOVA test, *p* p value for comparing between different groups, * Statistically significant at p ≤ 0.05

Melasma types, determined via Wood’s lamp, were predominantly epidermal (60%–66.7%) across all groups. The malar pattern was the most common distribution observed (60% in Group A, 53.3% in Group B, and 40% in Group C), followed by Centro-facial and mandibular patterns, without significant intergroup differences (p = 0.458). Disease duration ranged from 3 to 50 months and was comparable among the groups (p = 0.853). A positive family history of melasma was reported in 33.3% of Group A, 46.7% of Group B, and 20% of Group C (p = 0.301) Table 2.

**Table 2:** Melasma severity among the treated groups at different time points

	Group A (Metformin) (n = 15)	Group B (Tranexamic) (n = 15)	Group C (Kligman) (n = 15)	Test	P
Before				F = 0.106	0.900
Range	8.9 – 20.5	8.4 – 21.2	8.7 – 20.3		
Mean ± SD	14.16 ± 3.36	14.56 ± 4.17	14.82 ± 4.28		
2 weeks				F = 0.028	0.972
Range	8.9 – 20.4	7.6 – 20	8 – 19.9		
Mean ± SD	13.73 ± 3.12	13.55 ± 3.95	13.87 ± 4.17		
4 weeks				F = 1.136	0.331
Range	8.3 – 19.7	5.7 – 13.2	6.1 – 17		
Mean ± SD	11.62 ± 2.96	9.91 ± 2.74	10.91 ± 3.58		
6 weeks				F = 1.931	0.158
Range	8.2 – 19.5	5 – 12.1	5.8 – 16.6		
Mean ± SD	11.19 ± 3.03	9 ± 2.54	10.23 ± 3.51		
2 months				F = 3.234	0.049^*^
Range	7.5 – 19.1	4.3 – 11	5.2 – 16.2		
Mean ± SD	10.72 ± 3.12	7.91 ± 2.37	9.22 ± 3.48		
p1 = 0.015^*^, p2 = 0.182, p3 = 0.243					
Reduction percentage				F = 12.875	< 0.001^*^
Range	3.5 – 47.2	28.7 – 57.8	17.2 – 57.4		
Mean ± SD	22.11 ± 16.13	45.28 ± 9.29	38.21 ± 12.11		
p1 < 0.001^*^, p2 = 0.001^*^, p3 = 0.139					

*SD* Standard deviation, *F* one-way ANOVA test, *p* value for comparing between different groups, *p1* p value for comparing between group A and group B, *p2* p value for comparing between group A and group C, *p3* p value for comparing between group B and group C, * Statistically significant at p ≤ 0.05

### MASI score reduction

Baseline mMASI scores were similar across groups (Group A: 14.16 ± 3.36, Group B: 14.56 ± 4.17, Group C: 14.82 ± 4.28; p = 0.900). After 8 weeks of treatment, a significant reduction in mMASI was observed in all groups. Group B (TXA) showed the most marked improvement, with a post-treatment mean mMASI of 7.91 ± 2.37, compared to 10.72 ± 3.12 in Group A and 9.22 ± 3.48 in Group C. The difference among the groups was statistically significant (p = 0.049) Table 2.

**Fig. 1:**
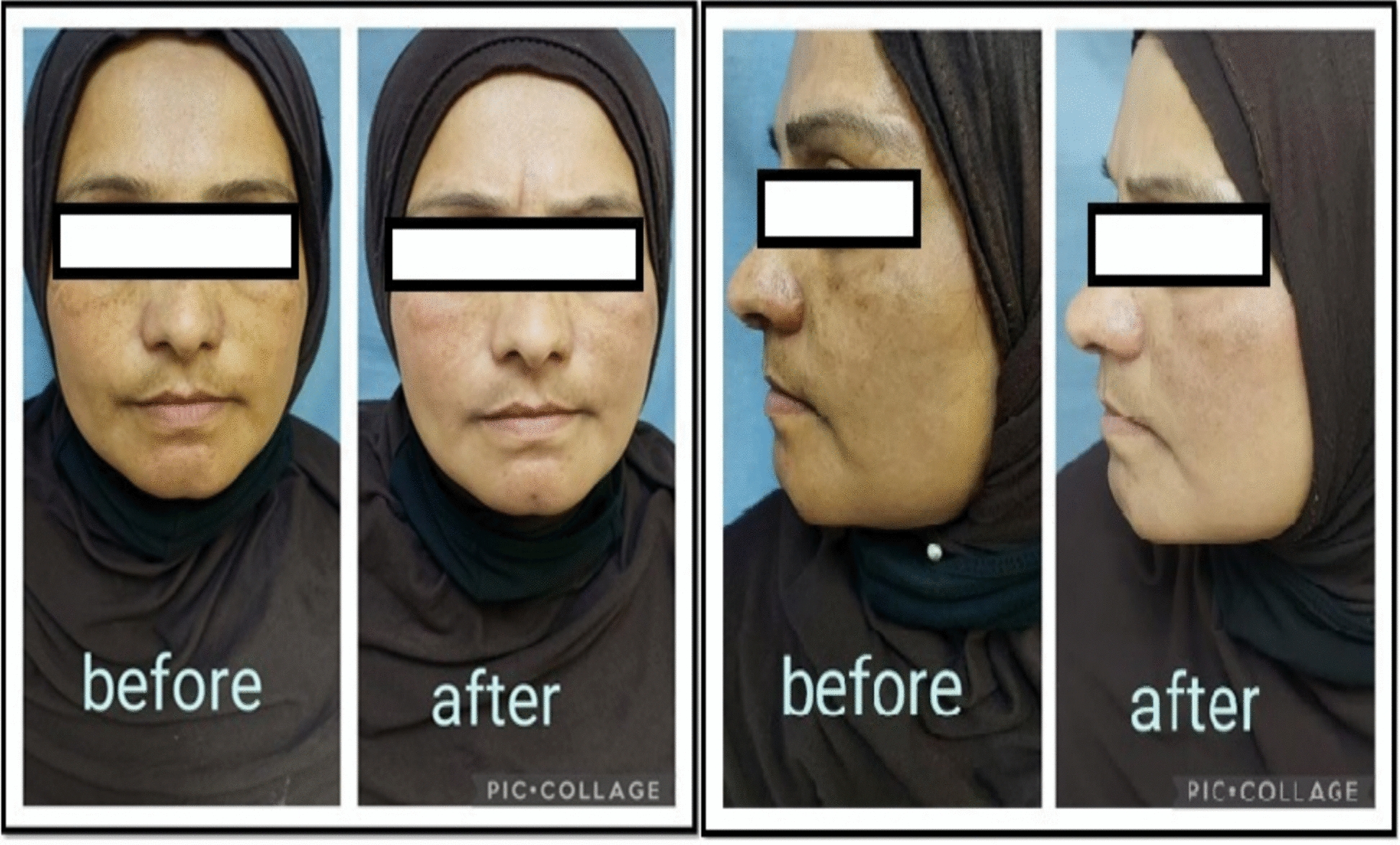
Frontal and lateral views of a patient treated with microneedling followed by topical tranexamic acid application. Before treatment: Moderate-to-severe facial hyperpigmentation, predominantly malar and mandibular in distribution. Simulated baseline mMASI score: 14.5. After 8 weeks: Marked improvement in pigmentation density and area of involvement, with increased skin luminosity and reduced perifollicular accentuation. Simulated post-treatment mMASI score: 7.5. Overall improvement is consistent with high patient satisfaction and a reduction percentage of approximately 48%

**Fig. 2:**
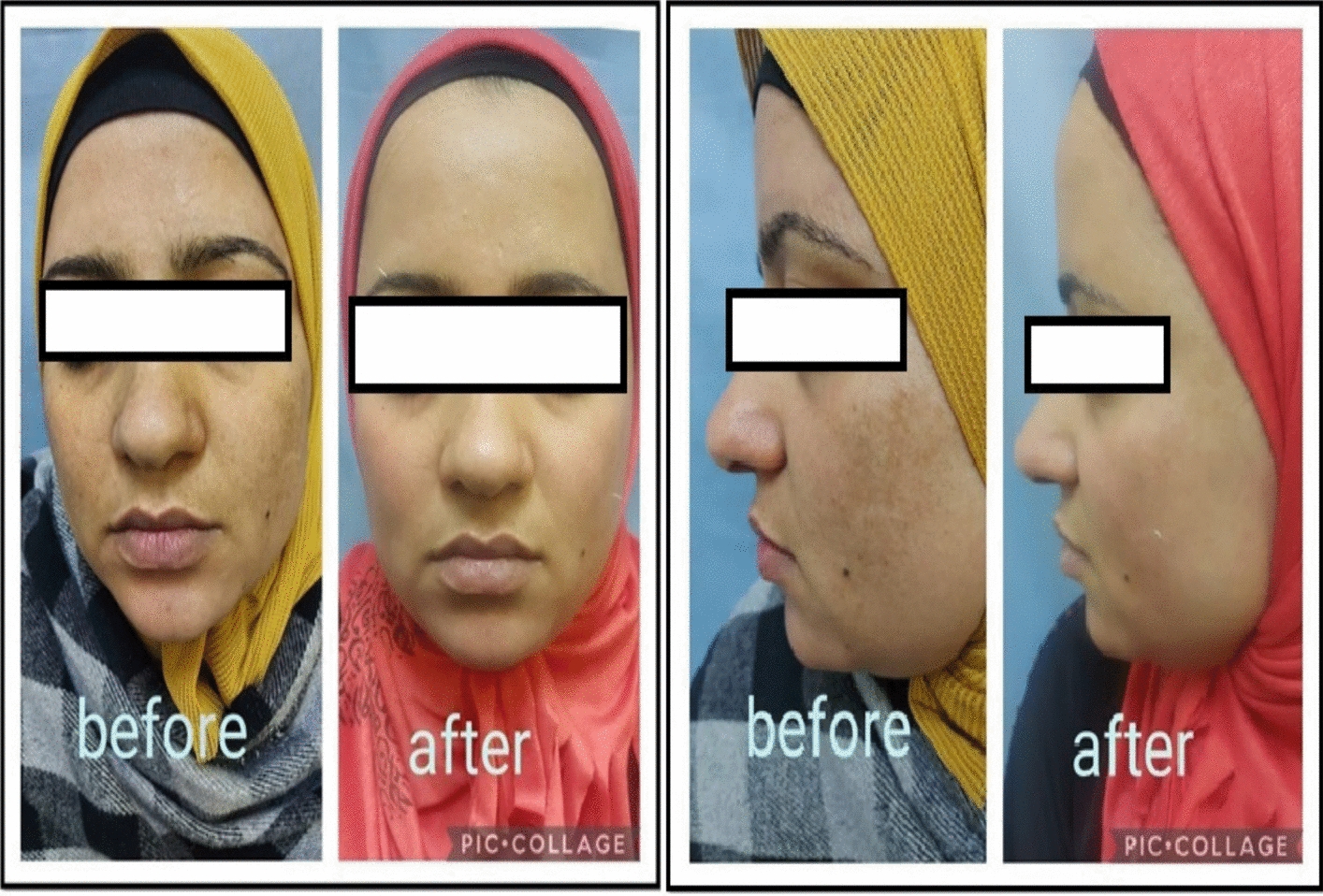
Frontal and lateral views of a patient treated with microneedling followed by topical metformin application. Before treatment: Widespread blotchy hyperpigmented macules across malar and centrofacial areas. Simulated baseline mMASI score: 15.0. After 8 weeks: Moderate reduction in pigment intensity and area. Improvement is visible but less pronounced compared to tranexamic acid group. Simulated post-treatment mMASI score: 11.2. Corresponds to a reduction percentage of approximately 25%, reflecting a modest clinical response

The mean percentage reduction in mMASI was highest in Group B (45.28 ± 9.29%), followed by Group C (38.21 ± 12.11%), and lowest in Group A (22.11 ± 16.13%). Group B significantly outperformed Group A (p < 0.001) and also had higher reductions compared to Group C, although this difference did not reach statistical significance (p = 0.139) Figs 1–2.

### Effect of skin type on response

There were no statistically significant differences in mMASI reduction based on Fitzpatrick skin type within any group. In Group B, type III and IV patients had mean mMASI reduction percentages of 47.52 ± 8.81% and 41.92 ± 9.75%, respectively (p = 0.267), suggesting consistent efficacy across skin types. Similar findings were noted in Groups A and C Table 3.

**Table 3:** mMASI score changes by skin type across and within treatment groups

Skin type	Group	Baseline mMASI (mean ± SD)	8-week mMASI (mean ± SD)	% Reduction (mean ± SD)	p-value (within group)	p-value vs. Group A	p-value vs. Group B	p-value vs. Group C
Type III	A	14.24 ± 3.24	10.17 ± 2.18	25.00 ± 16.27	0.344	–	< 0.001*	0.001*
Type III	B	16.04 ± 3.87	8.29 ± 1.87	47.52 ± 8.81	0.267	< 0.001*	–	0.197
Type III	C	15.21 ± 3.81	8.94 ± 2.63	41.04 ± 10.27	0.418	0.001*	0.197	–
Type IV	A	14.00 ± 3.96	11.82 ± 4.59	16.32 ± 15.87	0.344	–	< 0.001*	0.001*
Type IV	B	12.33 ± 3.85	7.35 ± 3.08	41.92 ± 9.75	0.267	< 0.001*	–	0.303
Type IV	C	14.48 ± 4.88	9.46 ± 4.26	35.74 ± 13.71	0.418	0.001*	0.303	–

*mMASI modified Melasma Area and severity index; Within-group p-values* compare pre- and post-treatment changes for the same skin type. *Intergroup p-values* assess percentage reduction differences across treatments for the same skin type. Statistically significant comparisons (p < 0.05) are marked with an asterisk (*)

### Correlation analysis

Spearman’s rank correlation analysis was conducted to explore the relationships between key clinical parameters and treatment outcomes. A strong positive correlation was observed between the percentage reduction in mMASI scores and patient satisfaction (r =  + 0.72, p < 0.05), indicating that patients who experienced greater clinical improvement also reported higher satisfaction levels. Conversely, a weak but statistically significant negative correlation was found between disease duration and mMASI reduction percentage (r = −0.28, p < 0.05), suggesting that patients with more longstanding melasma tended to show less marked improvement Table 4.

**Table 4:** Correlations between key clinical and outcome variables

Variables	Baseline mMASI	% Reduction in mMASI	Disease duration	Skin type	Patient satisfaction
Baseline mMASI	**–**	**–0.12**	** + 0.08**	** + 0.04**	**–0.09**
% Reduction in mMASI	**–0.12**	**–**	–0.28*	**–0.06**	+ 0.72*
Disease duration	** + 0.08**	–0.28*	**–**	** + 0.10**	**–0.18**
Skin type (III = 1, IV = 2)	** + 0.04**	**–0.06**	** + 0.10**	**–**	**–0.07**
Patient satisfaction	**–0.09**	+ 0.72*	**–0.18**	**–0.07**	**–**

Spearman’s correlation coefficients are shown. *p < 0.05 considered statistically significant. *mMASI* modified Melasma Area and severity index

No significant correlations were found between baseline mMASI scores and percentage reduction (r = −0.12), nor between skin type and either treatment response (r = −0.06) or satisfaction (r = −0.07). These findings suggest that initial melasma severity and Fitzpatrick skin type did not meaningfully influence the efficacy of the treatment modalities used in this study.

### Patient satisfaction

Patient satisfaction mirrored the objective improvements. In Group B, 33.3% of patients reported “marked improvement” (≥ 75% improvement), and 66.7% were classified as “excellent responders” (≥ 50% improvement). In contrast, Group A had no patients with marked improvement and only 13.3% with excellent responses. Group C had moderate outcomes, with 20% reporting marked improvement and 66.7% reporting moderate improvement. The differences in satisfaction were statistically significant (p = 0.001).

### Pain and adverse events

Pain was reported only in Groups A and B, with mean scores of 4.3 ± 0.7 and 4.1 ± 0.6, respectively (p = 0.54). Group C experienced no procedural pain. All procedures were well tolerated. Mild erythema and transient burning were observed in the microneedling groups, resolving within 48–72 h without intervention. No cases of post-inflammatory hyperpigmentation, scarring, or infection were reported. Table 5

**Table 5:** Patient-reported satisfaction among treated groups

Patient satisfaction	Group A (Metformin) (n = 15)		Group B (Tranexamic) (n = 15)		Group C (Kligman) (n = 15)		Test	P
	No	%	No	%	No	%		
Grade 0	9	60.0	0	0.0	2	13.3	χ 2= 18.163	0.001*
Grade 1	6	40.0	10	66.7	10	66.7		
Grade 2	0	0.0	5	33.3	3	20.0		

*IQR* interquartile range, *χ*^*2*^ Chi square test, *H* Kruskal Wallis test, *p* p value for comparing between different groups; *Statistically significant at p ≤ 0.05

### Dermoscopy findings

**Fig. 3:**
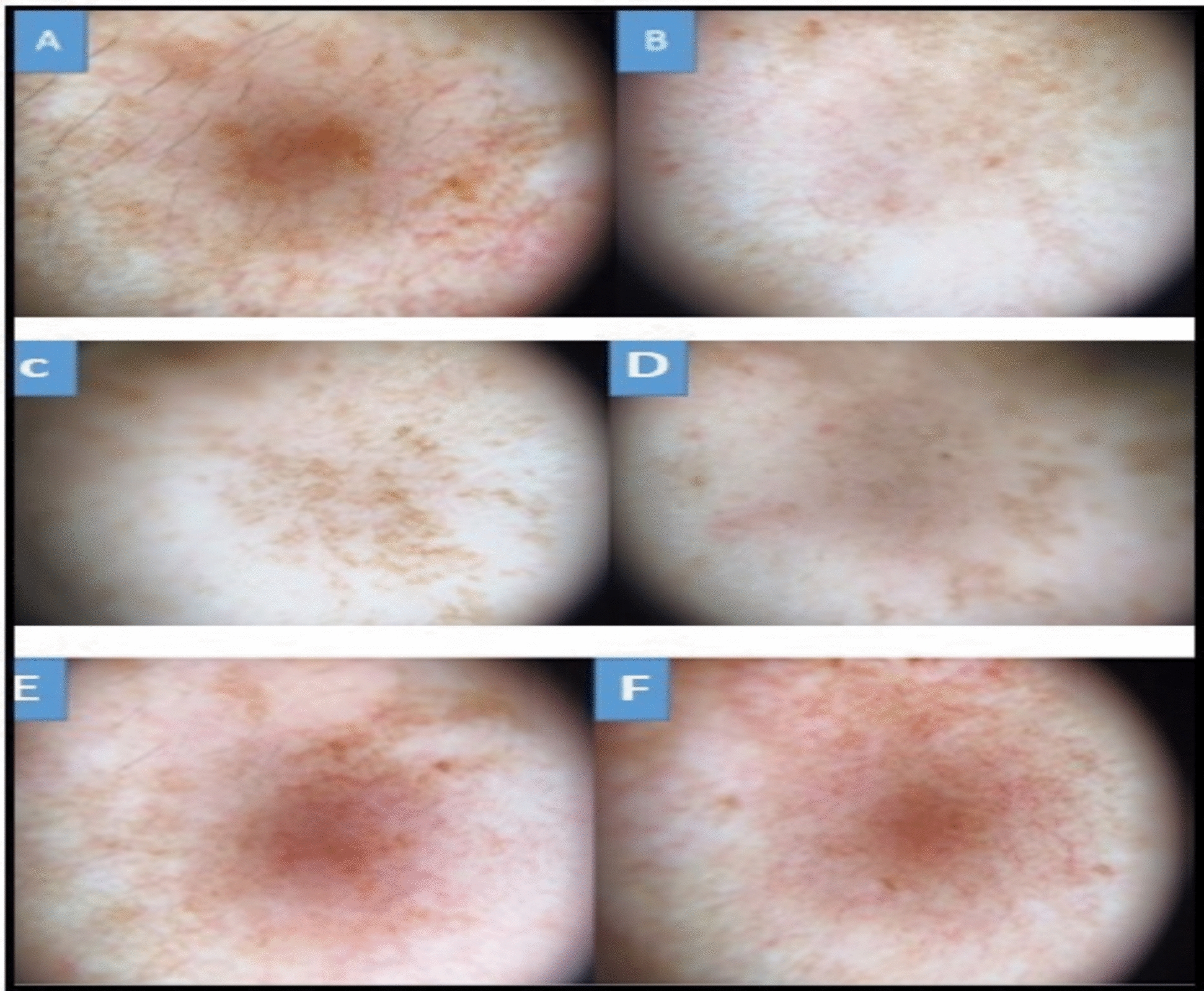
Polarized dermoscopic images showing melasma lesions before (**A**, **C**, **E**) and after (**B**, **D**, **F**) 8 weeks of treatment. **A**–**B** Group A – Microneedling with Metformin: Reduction in pigmentation density, thinning of the reticulated network, and improved skin homogeneity. **C**–**D** Group B – Microneedling with Tranexamic Acid (TXA): Marked clearance of pigmentation, near-complete flattening of the network, and uniform background tone. **E**–**F** Group C – Kligman’s Formula: Moderate improvement with lighter pigment background and partial reduction in vascular features

Dermoscopy revealed a significant reduction in pigment granularity and brown reticular network in Group B. Group C showed moderate improvements, while Group A had minimal dermoscopic changes. These findings corroborated clinical and patient-reported outcomes Figure 3.

## Discussion

Melasma continues to pose a therapeutic challenge due to its chronicity, multifactorial etiology, and frequent recurrences [2]. Our study aimed to assess and compare the efficacy and safety of microneedling-assisted delivery of topical metformin and tranexamic acid (TXA), two agents with emerging roles in pigmentation management, alongside a conventional topical Kligman’s formula group.

The results demonstrated that microneedling with TXA significantly outperformed both metformin and Kligman’s regimen in reducing modified MASI scores and improving patient satisfaction. These findings support the hypothesis that TXA, when delivered transdermally through microneedling, penetrates more effectively and produces superior clinical outcomes.

TXA has been increasingly explored as a depigmenting agent, attributed to its inhibition of plasminogen activation which reduces melanocyte-stimulating factors such as prostaglandins and arachidonic acid derivatives [5, 6]. Additionally, TXA inhibits angiogenesis by downregulating vascular endothelial growth factor (VEGF), which is particularly relevant given the increased vascularization in melasma lesions [7]. Our findings align with previous studies demonstrating significant MASI score reductions and better patient-reported outcomes with TXA delivered via microneedling [8–10].

In contrast, while metformin also exhibited melanogenesis-inhibiting effects, its clinical efficacy was comparatively modest in our cohort. Metformin is believed to exert its anti-melanogenic effects via the AMP-activated protein kinase (AMPK) pathway, leading to downregulation of MITF and related tyrosinase proteins [11]. Experimental studies have shown its depigmenting potential in vitro and in animal models [12, 13]. However, human data remains limited, and to our knowledge, our study is among the first to evaluate its topical use post-microneedling in melasma patients. The comparatively lower efficacy of metformin observed here may relate to its pharmacodynamic properties or formulation-based differences in skin absorption.

The use of Kligman’s formula, long regarded as a gold standard topical regimen, demonstrated intermediate results. Despite its known efficacy through the combined actions of hydroquinone (a tyrosinase inhibitor), tretinoin (a keratinocyte turnover promoter), and corticosteroids (anti-inflammatory agents), concerns over irritation, rebound hyperpigmentation, and skin atrophy limit its long-term application [14, 15]. Although mid-potency corticosteroids such as mometasone are generally avoided for prolonged use on facial skin due to risks of atrophy and hypopigmentation, its inclusion here reflects a locally compounded version of Kligman’s formula. Mometasone was chosen due to its balance of anti-inflammatory efficacy and tolerability in short-term regimens. In our study, no significant adverse effects were observed, likely due to short-duration use and strict post-treatment photoprotection, yet the improvement plateaued earlier compared to the TXA group.

Interestingly, treatment responses did not differ significantly across Fitzpatrick skin types III and IV, reinforcing the notion that both microneedling and the tested agents are safe and potentially effective across darker skin types, which are typically more prone to post-inflammatory hyperpigmentation [16]. This is particularly encouraging for clinical practice in regions with predominantly higher skin phototypes. Our findings revealed a weak but statistically significant inverse correlation between melasma duration and treatment response (r = −0.28, p < 0.05), indicating that patients with longer-standing melasma may show less pronounced improvement. This is consistent with observations from prior studies suggesting that chronic melasma is more likely to have dermal involvement, melanocyte hyperactivity, and disruption of the basement membrane, all of which may reduce treatment responsiveness [17, 18]. While not definitive, this relationship underscores the potential importance of early intervention and the need for further longitudinal studies to delineate the impact of disease chronicity. The vehicle differences between the metformin cream (PEG-based) and TXA solution (aqueous) may have influenced transdermal absorption and treatment efficacy. Creams may have slower or less efficient delivery compared to low-viscosity solutions during microneedling.

Microneedling, employed as a delivery enhancer, offers several mechanistic advantages. It induces micro-injuries that increase skin permeability and enhances transdermal drug delivery, bypassing the stratum corneum barrier [19]. Moreover, it promotes dermal remodeling, increases collagen production, and may help normalize the melanogenic activity of basal melanocytes by modulating their microenvironment [20]. These synergistic effects likely contributed to the favorable responses observed in both experimental groups.

Recent studies have expanded the understanding of microneedling-assisted delivery in melasma. Kuster Kaminski Arida et al. conducted a split-face study and found that microneedling alone achieved significant improvement in melasma severity, but adding tranexamic acid (TXA) did not provide additional clinical benefit [21]. In contrast, a meta-analysis by Poostiyan et al. concluded that the combination of microneedling and TXA yields superior outcomes compared to monotherapies [22]. Furthermore, Rajabi et al. compared microneedling-assisted TXA delivery with Q-switched Nd:YAG laser-assisted TXA, demonstrating comparable efficacy between both methods, though microneedling was associated with a longer downtime [23]. Additionally, Basu et al. showed that microneedling-assisted delivery of TXA and vitamin C both effectively improved melasma, with no significant difference between the two agents [24]. Recent evidence by Shabaan et al. [25] demonstrated superior outcomes using microneedling-assisted topical metformin versus vitamin C in a split-face trial. Their results align with our modest but positive metformin group response, supporting its emerging role as a melanogenesis inhibitor Table 6.

**Table 6:** Previous studies on microneedling and topical therapies for melasma

Author	Intervention	Comparator	Sample size	Main findings / conclusion
Zaky et al. [3]	Microneedling + TXA 4%	4% HQ)	30 (split-face)	Greater mMASI reduction with TXA microneedling vs HQ; microneedling + TXA effective, safe alternative
Aghdam et al. [8]	Microneedling + TXA + Kligman's formula	TXA alone, Kligman alone	120	Combination therapy showed superior efficacy; combination therapy most effective
Budamakuntla et al. [9]	TXA microneedling vs TXA microinjection	None	60	Both groups showed improvement; microneedling slightly better; TXA delivery methods effective
El-Husseiny et al. [10]	TXA 5% cream	HQ 4% cream	40 (split-face)	Similar efficacy; fewer side effects with TXA; TXA cream a good alternative to HQ
Lima et al. [18]	Microneedling alone	No treatment	10	Moderate improvement in melasma severity; microneedling may stimulate pigment lightening
Arida DKK et al. [20]	Microneedling + TXA vs Microneedling alone	None (split-face)	20	Microneedling alone effective; TXA added no significant benefit; Microneedling effective
Poostiyan et al. [21]	Microneedling + TXA vs Monotherapy	Meta-analysis	Various (12 RCTs)	Combination therapy superior to monotherapy; microneedling + TXA favored
Rajabi et al. [22]	Microneedling + TXA vs Laser + TXA	Microneedling vs Q-switched Nd YAG laser	60	Both groups improved; microneedling had longer downtime; Both combinations effective
Basu et al. [23]	Microneedling + TXA vs Microneedling + Vitamin C	None	30	Both combinations effective; no major difference; Both safe and effective
Shabaan et al. 2 [4]	Microneedling + Metformin vs Microneedling + Vitamin C	None (split-face)	30	Metformin group showed greater clinical improvement; metformin promising agent

* TXA* Tranexamic Acid, *HQ* Hydroquinone, *RCTs* Randomized Controlled Trials, *mMASI* Modified Melasma Area and Severity Index

Pain levels were mild and well tolerated, consistent with prior studies suggesting that microneedling, when appropriately performed with adequate topical anesthesia, is a safe and comfortable procedure for most patients [21]. Post-procedure erythema was transient and resolved without intervention, with no cases of infection, scarring, or pigmentary worsening reported.

## Limitations

This study has some limitations. First, the sample size was relatively small, which may restrict the generalizability of the findings. Second, the duration of follow-up was limited to 1-month post-treatment. Given melasma’s relapsing nature, longer-term data would be essential to evaluate sustained efficacy and recurrence rates. Third, while the formulation of topical metformin was standardized in-house, the pharmacokinetics of its dermal absorption were not evaluated, potentially contributing to the relatively modest clinical effects observed.

Furthermore, histopathological or immunohistochemical assessments (e.g., MART-1 or tyrosinase expression) were not conducted, which could have provided mechanistic insights and more objective biomarkers of response. While the mMASI score is a validated semi-objective clinical tool, more quantitative assessment methods (e.g., reflectance spectrophotometry, colorimetry, Antera 3D) were not used and may enhance precision in future studies.

This was a pilot study, and no a priori sample size calculation was performed. The modest sample size (n = 15 per group) increases the risk of Type II error. Lastly, the lack of a metformin-only or microneedling-only control arm limits our ability to distinguish between the pharmacologic and procedural effects of the interventions.

## Conclusion

In conclusion, this trial provides evidence that microneedling-assisted tranexamic acid delivery is significantly more effective than microneedling with metformin or topical Kligman’s regimen in reducing melasma severity and enhancing patient satisfaction. While topical metformin demonstrated modest efficacy, further research is needed to optimize its formulation and dosing for cutaneous use.

Microneedling emerges as a safe and effective adjunct for transdermal delivery in melasma management, particularly in darker-skinned populations. Future studies should include larger cohorts, longer follow-up, and incorporation of histological endpoints to better elucidate the mechanisms of action and sustainment of results.

## Data Availability

The data that support the findings of this study are available from the corresponding author upon reasonable request.

## Abbreviations

mMASI: Modified melasma area and severity index
TXA: Tranexamic acid
AMPK: AMP-activated protein kinase
MITF: Microphthalmia-associated transcription factor
SPF: Sun protection factor
VEGF: Vascular endothelial growth factor
PIH: Post-inflammatory hyperpigmentation
RCTS: Randomized controlled trials
SD: Standard deviation

## Code availability

All authors confirm all data and materials as well as software applications or custom code support their published claims and comply with field material.
